# Optimizing the precision of laser speckle contrast imaging

**DOI:** 10.1038/s41598-023-45303-z

**Published:** 2023-10-20

**Authors:** Alberto González Olmos, Sharvari Zilpelwar, Smrithi Sunil, David A. Boas, Dmitry D. Postnov

**Affiliations:** 1 Department of Clinical Medicine, Aarhus University, 8200 Aarhus, Denmark; 2 Department of Biomedical Engineering, Boston University, Boston, MA 02215 USA

**Keywords:** Imaging and sensing, Blood flow, Biomedical engineering

## Abstract

Laser speckle contrast imaging (LSCI) is a rapidly developing technology broadly applied for the full-field characterization of tissue perfusion. Over the recent years, significant advancements have been made in interpreting LSCI measurements and improving the technique’s accuracy. On the other hand, the method’s precision has yet to be studied in detail, despite being as important as accuracy for many biomedical applications. Here we combine simulation, theory and animal experiments to systematically evaluate and re-analyze the role of key factors defining LSCI precision—speckle-to-pixel size ratio, polarisation, exposure time and camera-related noise. We show that contrary to the established assumptions, smaller speckle size and shorter exposure time can improve the precision, while the camera choice is less critical and does not affect the signal-to-noise ratio significantly.

## Introduction

Laser speckle contrast imaging (LSCI) provides a rapid qualitative characterization of the motion of light-scattering particles. Over the past 20 years, it has become widely used as an imaging tool to measure blood flow in the brain^1–6^, skin^7–9^, retina^10–12^ and other organs. LSCI has had a broad impact on fundamental research, for instance, enabling the connection between migraine aura and headache^2^, and has a rising number of applications in human studies^13,14^.

The technique quantifies the speckle pattern blurring caused by the motion of scattering particles by calculating speckle contrast (*K*)^15^. The speckle contrast is defined by the exposure time (T) and the intensity autocorrelation function $g_2(\tau )$^16–18^. The intensity autocorrelation function, in turn, is related to the field correlation function $g_1(\tau )$ and, thus, to the decorrelation time $\tau _c$, a quantitative measure of the particles’ dynamics^15,17,19,20^. Over the past years, significant advances have been made to elucidate the relationship between speckle contrast and the decorrelation time, improving the interpretation and accuracy of laser speckle contrast imaging. It includes characterizing and correcting speckle-size and pixel-size related bias^21–23^, developing multi-exposure speckle imaging^18,19^, dynamic light scattering imaging^20^, exploring the potential errors caused by choice of the light scattering model^16^ and re-assessing the contrast calculation and the effects of static scattering^24–26^. These advances are crucial for steady-state blood flow monitoring, enable physiologically realistic thresholds for stroke imaging and open the way to quantitative LSCI measurements^20^.

On the other hand, evaluating fast temporal dynamics of the blood flow relies more on high precision than accuracy. Relevant applications cover all kinds of vascular responses and vasoreactivity, including functional activation, vasomotion and cardiac pulsatility. Nevertheless, the precision and signal-to-noise ratio aspects of LSCI are scarcely studied and have to be re-evaluated according to the modern LSCI theory and hardware. This study combines simulation, theory and animal experiments to systematically re-analyse the effects of the key factors defining LSCI precision—speckle-to-pixel size ratio, polarisation, exposure time and camera-related noise. Kirkpatrick et al. suggested that speckles have to be at least twice as large as pixels to maximise the contrast and, therefore, the signal-to-noise ratio in LSCI^21^. Later, Qiu et al.^27^ questioned this conclusion, showing that a smaller speckle size might benefit the LSCI precision. Our results confirm the latter and show how the error in the contrast measurements grows with the speckle size increase, reflecting the effect of insufficient speckle statistics. Note—we avoid calling it “speckle-noise” to avoid confusion with studies where speckle noise and speckle pattern are considered synonyms. Similarly to the speckle-to-pixel size ratio, polarising the detected light was assumed to be critical for the signal-to-noise ratio as it increases the contrast range^28^. However, we show that despite the increase in the maximum contrast, light polarisation does not significantly affect LSCI precision except for reducing visual artefacts caused by specular reflections. Yuan et al.^29^ have estimated the optimal exposure time needed to maximise the signal-to-noise ratio in brain perfusion imaging and found it to be approximately 5 ms. It became the most common exposure time for the LSCI experiments, although longer exposure times are used in some applications^30^. Here, we revise the effects of the exposure time on the LSCI precision using the updated contrast models, which have changed substantially^16,18^ since the study by Yuan et al. We show that in realistic settings, the optimal exposure time is generally shorter than 5 ms, as longer exposure times eventually cause a decrease in absolute and relative contrast sensitivity and increase the error caused by insufficient speckle statistics. Finally, we show that the effect of the camera-related noise is negligible compared to the impact of insufficient speckle statistics, making it nearly impossible to improve the signal-to-noise ratio by using more expensive cameras.

## Results

### The principal role of insufficient speckle statistics

**Figure 1:**
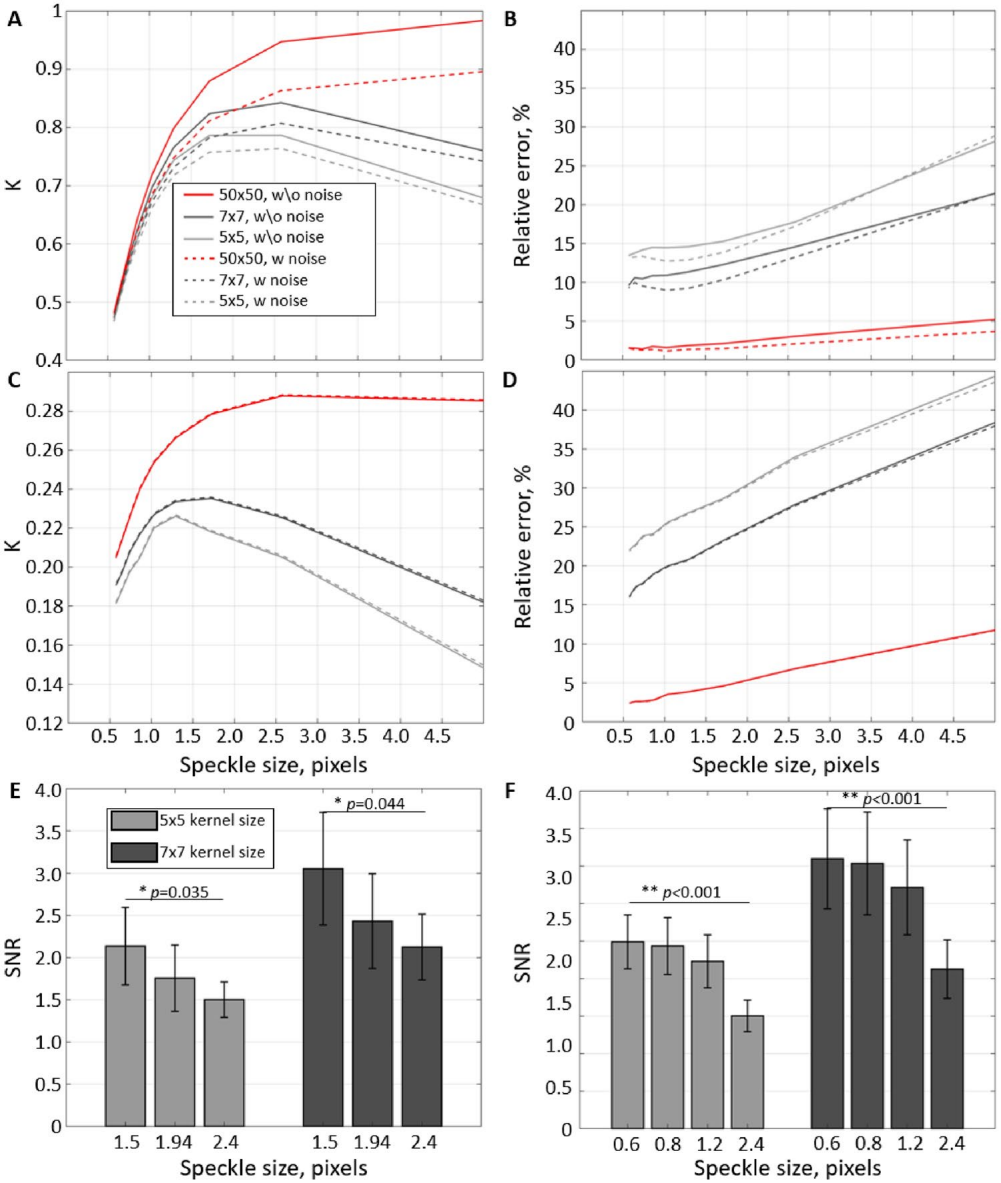
The principal role of speckle statistics. (**A**–**D**) Mean contrast *K* and the corresponding relative error estimated from the simulation using independent frames with $T/\tau _c=0$ (**A**,**B**) and $T/\tau _c=10$ (**C**,**D**). Lines colour reflects the pixel’s neighbourhood size used in the contrast calculation. Solid lines correspond to simulations where the error is defined only by speckle statistics, and dashed lines to simulations with the camera-related noise enabled. (**E**,**F**) Signal-to-noise ratio (SNR) measured from the experimental data for different speckle to pixel sizes. (**E**) Speckle size was adjusted physically by closing or opening the iris. (**F**) Speckle size was changed by numerically binning the pixels in recordings with the original speckle-to-pixel size ratio of 2.4. Both in simulation (**B**,**D**) and experimental data (**E**,**F**), it is clear that a decrease in the number of speckles per contrast neighbourhood leads to a reduction in the precision of the measurements. At the same time, the effect of camera noise and data discretization (dashed lines **B**,**D**) appears negligible compared to the importance of speckle statistics.

LSCI relies on estimating the contrast of the speckle pattern in a limited-size pixel’s neighbourhood (typically 5 × 5 or 7 × 7 pixels). Such estimation would depend on the speckle statistics—the number of sampling points (pixels) per speckle and the number of speckles in the neighbourhood. Insufficient statistics (low number of sampling points or speckles) will cause a reduction in accuracy (bias) and precision (increased measurement error). Kirkpatrick et al.^21^ have shown that the contrast can reach the theoretical maximum only when the speckle-to-pixel size ratio is larger than 2 and the pixel’s neighbourhood is sufficiently large. A decrease in the speckle-to-pixel size ratio or in the neighbourhood size will lead to underestimating the contrast (Fig. 1A). Although this bias can be mathematically corrected^22^, the increased measurement variation caused by insufficient speckle statistics can not. Figure 1B shows how the per-pixel (or per-measurement) error in the contrast estimation changes for different neighbourhood and speckle sizes. With the speckle size increase from $\approx 0.5$ to 5, the error grows from $\approx$14 to 28% ($\approx$2 times) for 5 × 5 neighbourhood, and from $\approx$9 to 22% ($\approx$2.4 times) and from $\approx$1 to 5% ($\approx$5 times) for 7 × 7 and 50 × 50 neighbourhoods. It confirms that, even for large neighbourhoods, smaller speckles result in more precise measurements. Furthermore, although the camera-related noise strongly affects the accuracy of estimating contrast value, it has a minor effect on the precision (dashed lines Fig. 1A,B). The relative error increases even more when quantifying speckle fluctuations at $T/\tau _c>1$, while the camera-related noise effect becomes negligible. For $T/\tau _c=10$ (Fig. 1C,D), the relative error has increased by $\approx 50{-}150\%$ depending on the neighbourhood size (more for larger neighbourhoods). Interestingly the error growth speed as a function of speckle size has remained approximately the same—$\approx$2, 2.4 and 5 times for 5 × 5, 7 × 7 and 50 × 50 neighbourhoods, respectively.

The experimental results confirm the simulation prediction. Figure 1E shows the per-pixel signal-to-noise ratio (SNR) of the cardiac signal in the brain parenchyma when speckle size is adjusted by physically opening and closing the aperture. With the reduction of speckle size from 2.4 to 1.5 pixels, the average SNR has increased from $\approx$1.5 to 2.2 (by 46%) and from 2.2 to 3.1 (by 40%) for the 5 × 5 and 7 × 7 neighbourhoods. To reach an even smaller speckle-to-pixel size ratio and confirm the tendency, we have spatially decimated (box filtering followed by downsampling) the raw intensity images and repeated the analysis (Fig. 1F). The tendency persisted, leading to an improved per-pixel SNR for smaller speckle sizes. Overall the experimental results show that a twofold increase in the per-pixel signal-to-noise ratio of contrast measurements can be achieved by changing from a 5 × 5 neighbourhood size at the speckle-to-pixel ratio of 2.4 to a 7 × 7 neighbourhood size at the speckle-to-pixel size ratio of 1.5.

### The effects of polarisation on the image and signal quality

**Figure 2:**
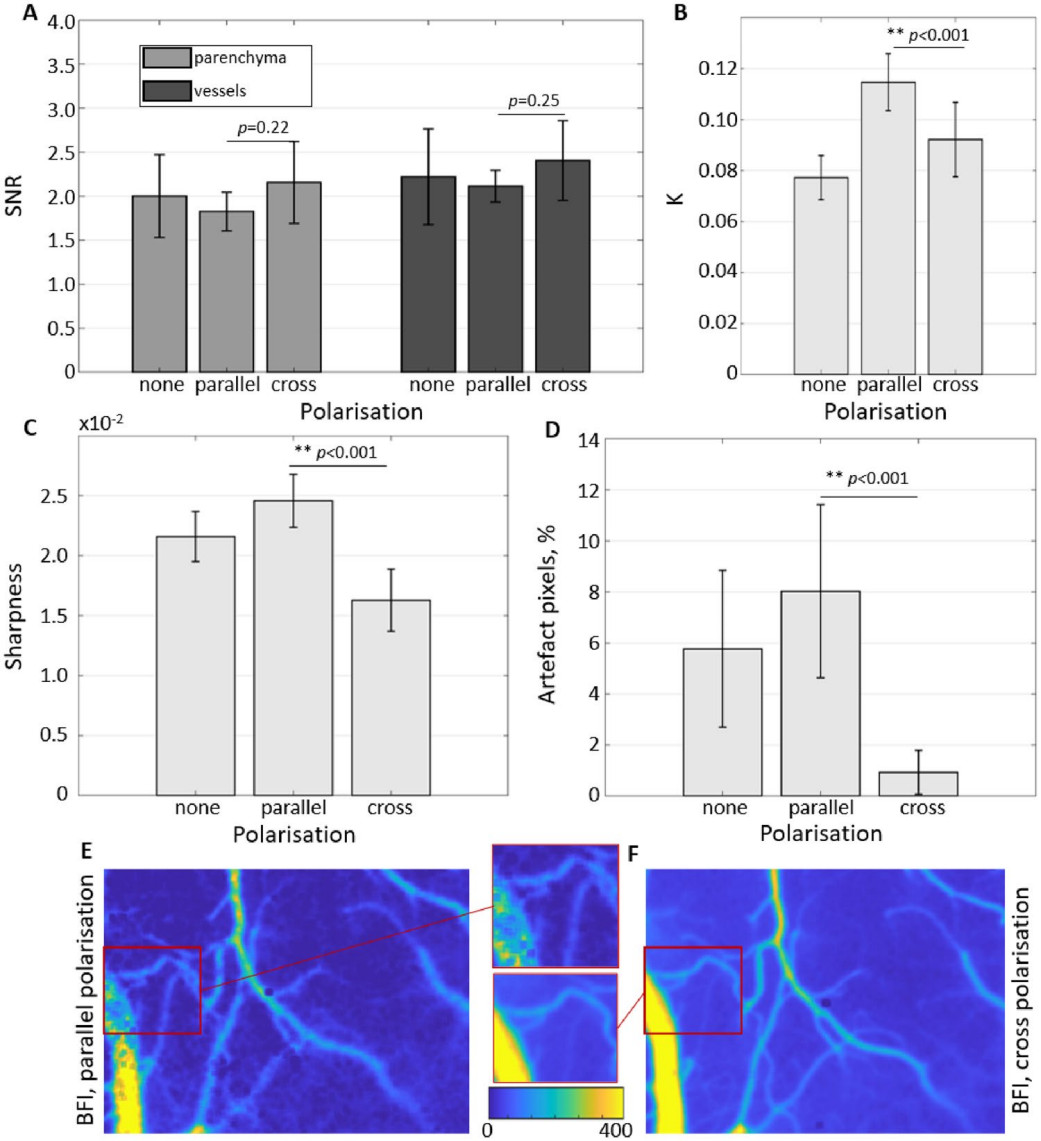
The effects of polarisation on the image and signal quality. (**A**) The signal-to-noise ratio of the cardiac signal in vessels and parenchyma measured without polariser, polariser in parallel orientation and polariser in cross orientation. (**B**) Contrast *K* averaged over the field of view. (**C**) Image sharpness averaged over the field of view. (**D**) Artefact pixels count relative to the total number of pixels in the field of view. (**E**,**F**) Exemplary blood flow index maps with polariser in parallel and cross orientations. Zoomed-in regions highlight the increased number of artefact pixels and, at the same time, the increased sharpness of the parallel orientation.

As the results above suggest that the contrast range has an insignificant role in typical LSCI applications, one would expect that polarisation of the detected light will also have a minimal effect on the signal-to-noise ratio. Data shown in Fig. 2A confirms this suspicion, with the SNR not changing significantly between the polariser configurations. It is happening despite the average contrast, and therefore the contrast range, changing as expected according to the theory (Fig. 2B). The average contrast is at its lowest when the polariser is removed ($<K>=0.077$) and is increased by 1.49 times ($<K>=0.115$) when the polariser is in the “parallel” configuration. It aligns well with the theoretical maximum of speckle contrast for non-polarised light being $1/\sqrt{2}$ times smaller than for polarised light^31^. The most commonly used “cross-polarised” configuration yields an average contrast of 0.092. The difference in the average contrast between the “parallel” and “cross” configurations can be explained by the latter leading to an increased number of multiple-scattering events and a reduced number of single-scattering events detected by the camera, which leads to reduced contrast values for the constant decorrelation time^16^. It might also explain a slight reduction in the SNR when using the “parallel” orientation, which is more noticeable in the parenchyma (15%) compared to the vessels (12%). Furthermore, it fits well with a significantly lower sharpness when “cross” polarising the detected light, which is decreased by $\approx 24\%$ and $34\%$ compared to polariser being removed or adjusted in “parallel” orientation, respectively (Fig. 2C). However, it is essential to note that both sharpness and SNR calculations exclude “artefact” pixels, which are majorly present in the absence of a polariser or its “parallel” but not in the “cross” orientation (Fig. 2D). The latter makes the “cross” the most preferential option overall. The exemplary images showing differences in sharpness and the presence of artefacts are shown in Fig. 2E,F.

### Defining the optimal exposure time

**Figure 3:**
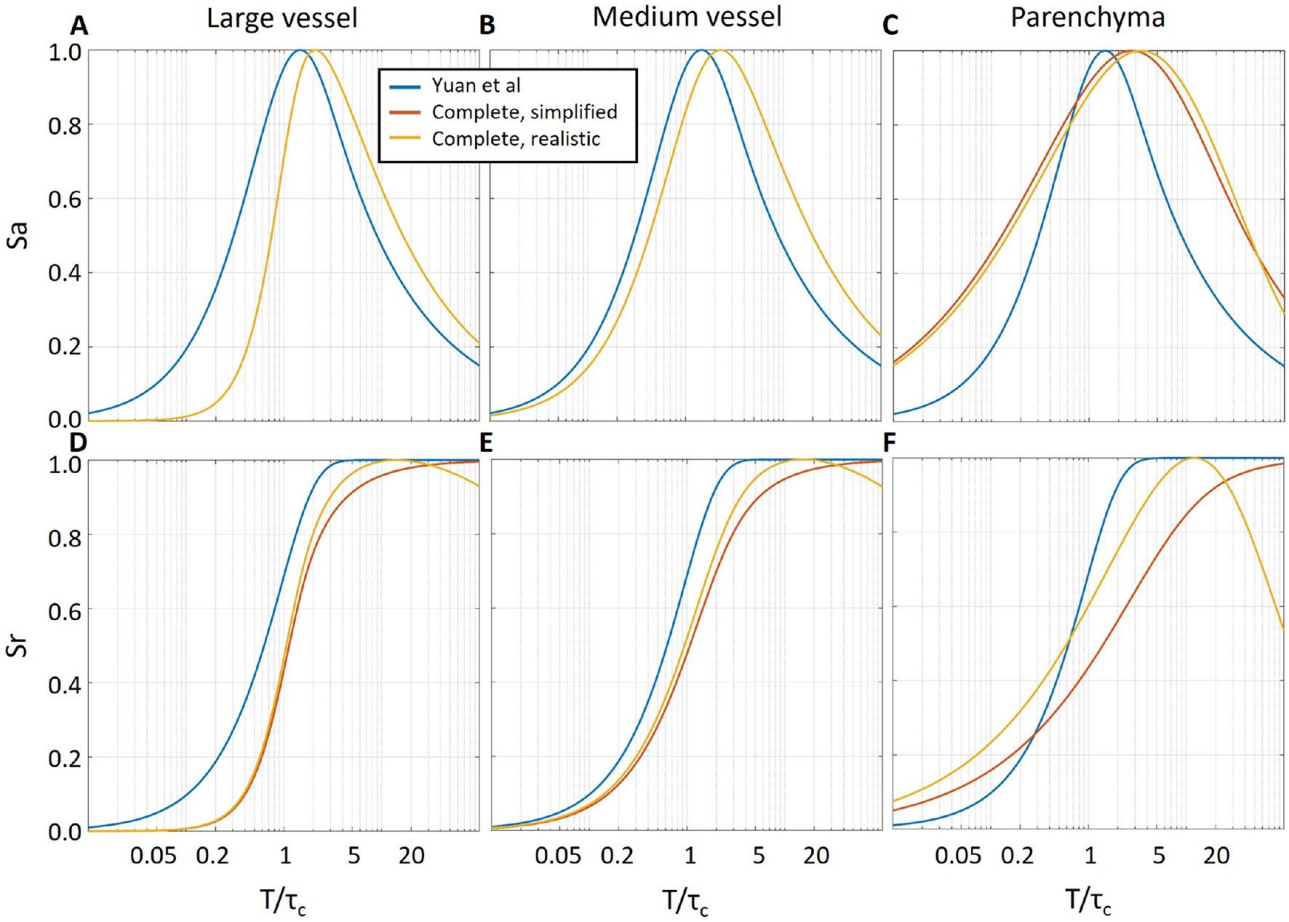
Effect of exposure time on the absolute (**A**–**C**) and relative (**D**–**F**) LSCI sensitivity to the flow change according to theoretical calculations. The blue lines correspond to the original derivations by Yuan et al.^29^, while red and yellow lines correspond to the calculations made with respective contrast models for large ($\tau _c=75 \; \upmu \text{s}$, Eq. (3)) and medium ($\tau _c=150 \; \upmu \text{s}$, Eq. (4)) vessels and parenchyma ($\tau _c=250 \; \upmu \text{s}$, Eq. (5)). For the simplified (red lines) model parameterization, the $\rho$ and *C* parameters were set to 1 and 0, independent of the simulated vessel type. For the realistic (yellow lines) model parameterization, *C* was set to 0.01 for all simulated vessel types, while $\rho$ was set to 1 in large and medium vessels and 0.8 in the parenchyma. It is vital to notice that not only the sensitivity curves are altered depending on the vessel type but that the presence of noise or static scattering ($C>0$ or $\rho <1$) leads to a decrease in the relative sensitivity after $T/\tau _C$ reaches the value of $\approx$10–15.

The optimal exposure time in LSCI is defined by the decorrelation time $\tau _c$, the contrast sensitivity and the combination of camera-related noise and speckle statistics. In Fig. 3, we show theoretical absolute $S_a=|\frac{dK}{dv}|$ and relative $S_r=|\frac{dK/K}{dv/v}|$ contrast sensitivity to the flow changes, where the flow speed *v* is proportional to $1/\tau _c$. Our calculations (yellow and orange lines) are using the recently derived and most complete contrast models, which differ depending on the dynamics regime in the region of interest (Eqs. 3–5). The results show that the exposure time at which the maximum sensitivity is reached is the shortest for the large vessels, increased for medium-sized vessels and further increased for the parenchyma. In all cases, relative and absolute sensitivity reaches the maximum at larger $T/\tau _c$ values than predicted by Yuan et al.^29^ (blue lines). Most importantly, when the contrast is offset either due to noise ($C>0$, relevant to all dynamic regimes) or due to static scattering (typically relevant only to parenchymal regions), the relative sensitivity does not saturate with increased exposure time but instead peaks at $T/\tau _c$ between 2 and 10 and then reduces. It has an important implication that increasing the exposure time will, at some point, lead to a decreased measurement sensitivity even when not accounting for the effects of speckle statistics or the camera-related noise.

**Figure 4:**
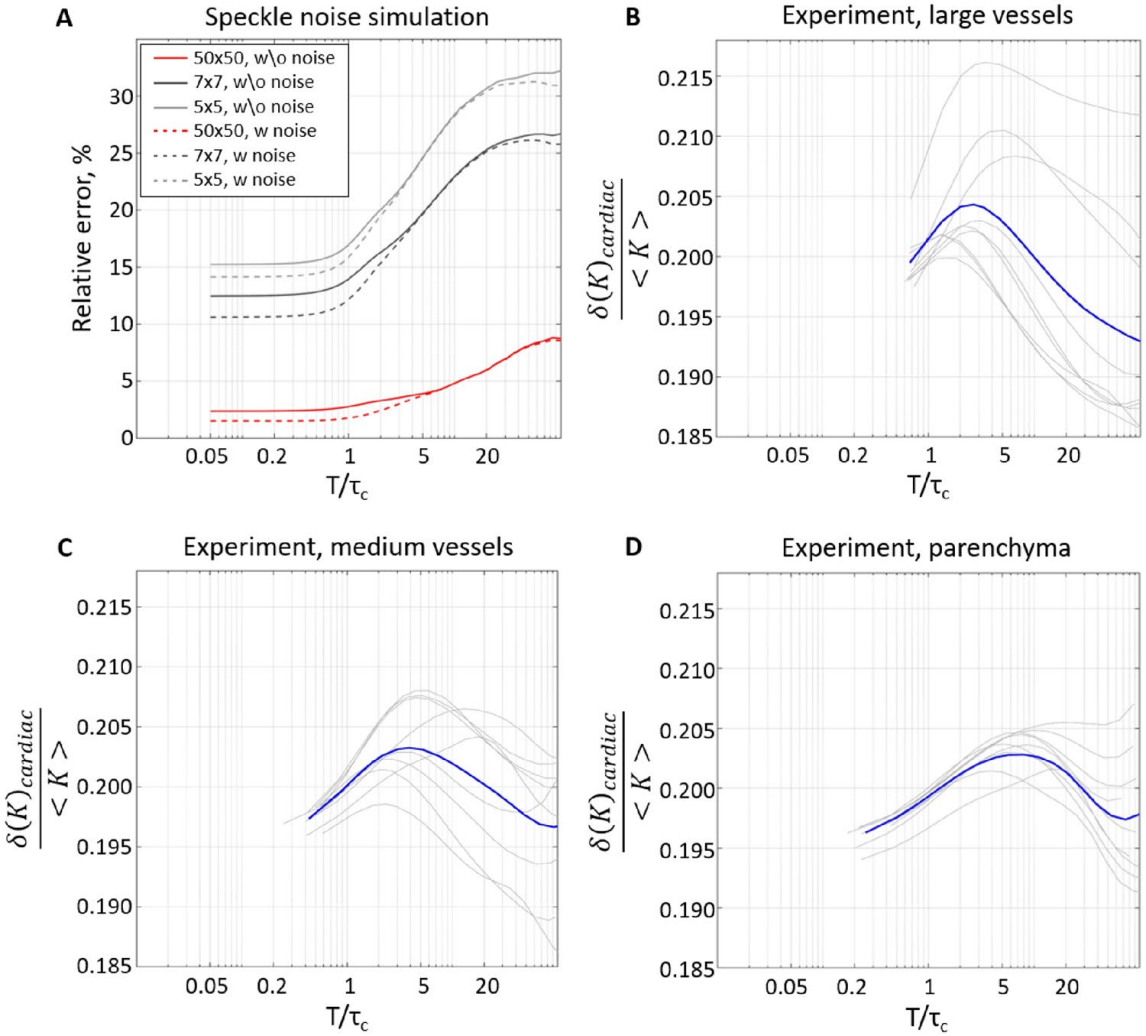
Effects of the exposure time on speckle noise and measurements precision. (**A**) Per-pixel relative error in contrast measurements, estimated from the simulated independent frames with $T/\tau _c$ ranging from 0.05 to 40. Lines colour reflects the pixel’s neighbourhood size used in the contrast calculation. Solid lines correspond to simulations where the noise is defined only by speckle statistics. Dashed lines correspond to simulations that additionally include a typical camera-related noise. (**B**–**D**) Experimentally measured contrast variation around the mean $\frac{\sigma (K)_{cardiac}}{<K>}$, which is dominated by cardiac pulsatility rather than noise. Grey lines show measurements from 3 animals, 3 regions of interest each. Blue lines show the average across all animals and regions of interest. In (**B**–**D**), larger values mean higher sensitivity to the physiological signal. Therefore, they confirm that the LSCI signal quality peaks at $T/\tau _c$ between 2 and 10.

Figure 4A shows how the error associated with the insufficient speckle statistics and the camera-related noise changes depending on the ratio between the exposure and decorrelation time according to the simulation results. When $T/\tau _c$ rises from 5 to 20, the per-pixel error increases 1.24, 1.31 and 1.5 times for the 5 × 5, 7 × 7 and 50 × 50 neighbourhoods, respectively. Similar to the results shown in Fig. 1, the speckle statistics play the most prominent role, while the camera-related noise has little effect. It reinforces the assumption that longer exposure time does not necessarily improve the contrast measurements’ reliability. Animal experiment results (Fig. 4B–D) confirm it by showing how the contrast variation caused by cardiac activity changes relative to its mean. This metric reflects the relative contrast sensitivity and, similar to the theoretical results, we see that the sensitivity is maximized when $T/\tau _c$ takes values from 2 to 10. Considering that for most LSCI systems and applications, the decorrelation time is in the range of 0.02–0.2 ms for vessels^19,20,24^ and 0.2–0.5 ms for capillaries^19,20,24^—we expect the optimal exposure time to be below 5 ms for parenchyma ($\approx 3$ ms), and as short as 0.1–1 ms in vessels.

### Choosing a camera for LSCI

**Figure 5:**
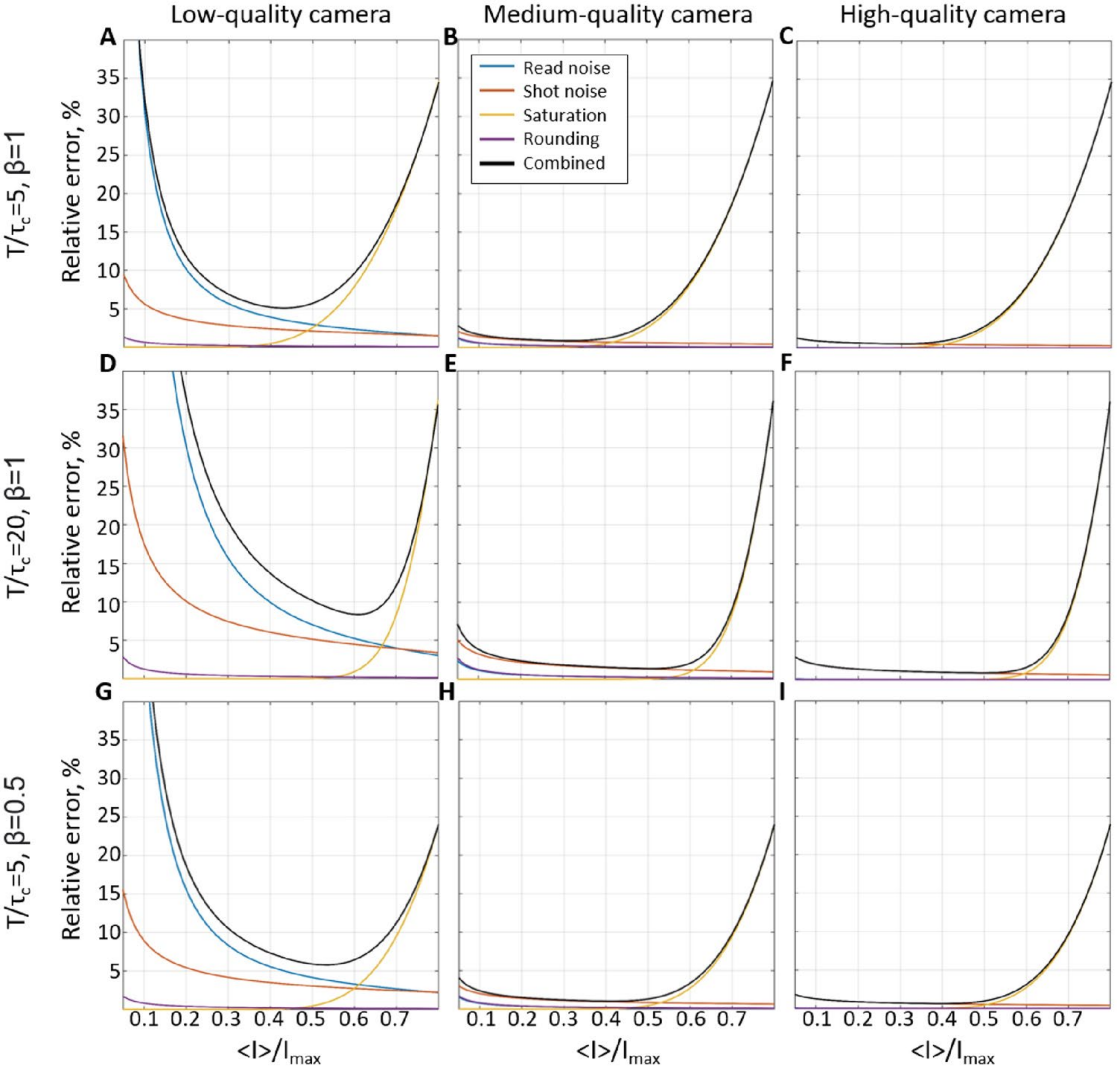
Effect of the camera-related noise on the per-pixel error relative to the “noise-free” contrast measurements. (**A**,**D**,**G**) Simulation results for a “low-quality” camera (QE = 0.2, 8 bit, $\sigma _r$  = 30$e^-$, saturation capacity 1200$e^-$) with the respective $T/\tau _C$ set to 5,20 and 5, and $\beta$ is set to 1, 1, and 0.5. (**B**,**E**,**H**) and (**C**,**F**,**I**) panels reflect the same simulations for a “medium-quality” camera (QE = 0.45, 8 bit, $\sigma _r$ = 13 electrons, saturation capacity 12,400$e^-$) and a “high-quality” camera (QE = 0.8, 16 bit, $\sigma _r$ = 1 electron, saturation capacity 30,000$e^-$). The lines’ colour reflects the type of noise or their combination.

In the sections above, we have observed that the camera-related noise appears to have little-to-no effect compared to the speckle statistics. It, however, might change depending on the camera quality, exposure time and the amount of light reaching the sensor. To identify the conditions in which the camera-related noise becomes significant and understand how the camera choice affects LSCI precision, we have simulated shot and read noise, saturation, and rounding effects on the speckle patterns. In Fig. 5, panels A, D, G correspond to a low-quality camera, B, E, H to a medium-quality camera (e.g. acA2040-90umNIR, Basler) and C, F, I to a high-quality scientific CMOS camera. The corresponding camera parameters were set as follows—quantum efficiency $QE=0.2$, 0.45 and 0.8, read noise standard deviation $\sigma _r=30$, 13 and $1e^-$, pixel bit-depth of 8, 8 and 16 bit, and saturation capacity 1200, 12,400 and 30,000$e^-$. The per-pixel error was calculated relative to the noise-free contrast (rather than to the mean contrast as in Fig 1) for the amount of light varying from 0 to total saturation capacity. For convenience, we display the error as a function of $<I>/I_{max}$, where $<I>$ is intensity averaged over the whole imaging and $I_{max}$ is a saturation value of the intensity (255 and 65,535 for 8 and 16 bit pixel-bit depth respectively), as these parameters, unlike flux, can be easily evaluated during the recording. The results show that for medium and high-quality cameras, the total error is below 2% when $I/I_{max}$ is between 0.2 and 0.4 (Fig. 5B,C). The error caused by the saturation (or value cut-off) rapidly grows for higher intensity values, while the shot noise slightly increases for the lower ones. The prevalent role of the saturation error is expected due to the wide distribution of speckle intensity, particularly for small $T/\tau _c$ and high coherence degree ($\beta \approx 1$). For larger $T/\tau _c$ or lower $\beta$, growth of the saturation error will be delayed as the speckle intensity distribution becomes more narrow. At the same time, it would lead to a more prominent effect of shot and read noises, which, nevertheless, only appears significant for a low-quality camera. In the case of medium and high-quality cameras, shot noise only becomes critical when the detected intensity is especially low, which is rarely the case for typical LSCI applications. Interestingly, rounding does not appear to play a significant role compared to other sources of error, implying that there is little benefit in using higher bit-depth values unless dealing with applications with a particularly low coherence degree or long exposure time. Overall the results suggest that for most LSCI applications, medium and high-quality cameras will have a similar performance, which will peak at $I/I_{max}\approx 0.4$ (e.g. $<I>=100$ for an 8-bit camera). It might be beneficial, however, to operate at even lower values of $I/I_{max}\approx 0.3$, as the optimal range might shift depending on the application or dynamics in the region of interest.

## Discussion

In this study, we have systematically evaluated the role of key factors affecting the precision of LSCI measurements. Unlike accuracy, the precision should not affect the interpretation of the average contrast but is crucial in allowing reliable characterization of fast dynamic changes and reducing the number of repeated measurements (e.g. during neurovascular coupling) and the overall number of animals required according to the power calculations. We have found that contrary to the conventional belief—the contrast range does not play a critical role in the LSCI signal-to-noise ratio for typical applications. Consequently, maximizing the contrast by having at least two pixels per speckle does not translate to noticeably increased precision. On the contrary, as the number of speckles in the contrast calculation neighbourhood is reduced for larger speckles, the precision decreases. The simulation results show that the relative per-pixel error in the contrast estimation seems to increase linearly with the speckle size at a rate that depends on the number of pixels in the neighbourhood (Fig. 1B,D). For the 5 × 5 pixels neighbourhood, increasing the speckle size by a factor of 10 led to a twofold increase in the error. For the 7 × 7 and 50 × 50 neighbourhoods, the respective increase was 2.4 and 5 times. Interestingly the error growth rate did not change for different $T/\tau _c$ ratios. The results were confirmed experimentally, showing a 46% increase in the signal-to-noise ratio when decreasing the speckle size from 2.4 to 1.5 pixels in a 5 × 5 neighbourhood. The SNR was further increased when using a larger, 7 × 7 neighbourhood. The latter, however, is to be expected as the number of pixels over which the contrast estimation is done has also increased. Analysis of the polarisation effect has reinforced the conclusion that speckle statistics are more important for the LSCI precision compared to the contrast range, as no improvement in the SNR was found when the detected light was polarised, despite the evident change in the maximum contrast (Fig. 2A,B). Nevertheless, using a “cross” polarisation configuration improves the image quality by significantly reducing the number of artefacts caused by specular reflections. The optimal exposure time evaluation has shown that the precision is maximized when $T/\tau _c$ takes a value from 2 to 10. Greater than that, it does not only lead to reduced absolute sensitivity, as predicted by Yuan et al. but also to a reduction in relative sensitivity and increased error. Finally, our simulation predicts that the contribution of the camera-related noise would be negligible for most cameras typically used in LSCI as long as the average recorded intensity is $\approx$30–40%. The saturation error rapidly increases for higher intensities unless the exposure time is long or the coherence degree is low. It is essential to note, however, that some of the LSCI applications or modifications might require using longer exposure times to counter low light intensity (e.g. retinal imaging^30^ or fibre-based LSCI^32^) or low-coherence light sources to allow confocal gating. For such applications, it is expected that the role of the camera-related noise will increase substantially and might require the use of scientific CMOS cameras.

To conclude the results described above, we recommend using speckle-to-pixel size ratios below 2, exposure time within 2–10 decorrelation times and the light intensity of $\approx 30\%$ to maximize LSCI precision and image quality. Our results not only show how one can improve the precision of LSCI measurements but also outline a key limitation and a potential field for future research in LSCI methodology. Specifically, we have shown that the speckle statistics, i.e. the number of speckles within the neighbourhood, is much more important to the LSCI precision than the camera quality. It means that, unlike 20 years ago, the development in the sensors technology is unlikely to bring a qualitative change in LSCI measurements SNR, and instead, other approaches to improve it must be found. While reducing the speckle size is the most obvious solution and can have additional benefits, such as homogenizing the per-pixel contribution of static scattering, it might be challenging to achieve. The speckle size is proportional to the F-number, so minimizing the speckle size will result in losing the depth of focus. Therefore to fully benefit from the small speckle size and truly maximize the technique’s precision, the ways to reject an out-of-focus signal (e.g. via coherence gating or light-sheet illumination) must be explored.

## Methods

### Simulation

Simulations of the speckle pattern evolution have been proven helpful in improving the interpretation of LSCI^33,34^ and understanding the effects of external factors, such as camera noise^32^ or laser quality^35^. In this study, we use our previously published speckle dynamics model^32^, where the speckle pattern evolution is defined by the motion of the particles rather than by a pre-defined correlation function. Briefly, a number of particles, each considered a source of an electric field, are randomly distributed in a three-dimensional volume of a chosen size. The electric fields from the particles superimpose on the camera sensor at some distance from the volume, generating a speckle pattern. The intensity *I* of each pixel of the sensor is calculated with the following equation:

1 $$\begin{aligned} I= |E|^2 = \left |\sum _{n=1}^{N} \frac{e^{i \cdot k \cdot r_{n}}}{r_{n}}\right |^2, \end{aligned}$$

where *E* is electric field, *k* is wavenumber ($k = 2\cdot \pi / \lambda$, where $\lambda$ = 785 nm) and $r_{n}$ is the distance between the particle *n* and a pixel. When calculated for each pixel, it results in an instantaneous frame with infinitely short exposure time (T = 0 μs). The dynamics are introduced by displacing particles according to a defined motion pattern between frames. Consecutive frames are generated at time intervals much shorter than the desired decorrelation time $\tau _c$ and are averaged to simulate images with the desired exposure time. The shot and read noise, discretization and saturation can then be introduced to the images to test the role of camera-related signal alteration^32^.

With the process described above, we generated two sets of dynamic speckle images. The first set we used to demonstrate the effects of the speckle size on the per-pixel contrast estimation error. It consisted of 20,000 instantaneous frames of 500 × 500 pixels with a speckle-to-pixel size ratio of 5. At this step, the speckle size was measured as full-width at half maximum of the spatial autocovariance function^21^. Then, the frames were low-pass filtered with a series of box-car convolution filters and downsampled to produce patterns with smaller speckle sizes^21^. The second set we used to demonstrate the effects of exposure time and camera-related noise, such as shot noise, read noise and discretization. It consisted of 100,000 instantaneous frames of 200 × 200 pixels with a speckle-to-pixel size ratio of 2. In both simulations, the particles moved in an ordered motion pattern with the interval between instantaneous frames of 1 μs and the decorrelation time $\tau _c=20 \; \upmu \text{s}$. The latter was validated by calculating the intensity autocorrelation function $g_2(\tau )$ and fitting it with the single scattering ordered motion dynamic light scattering model^20^. Unless mentioned otherwise, the camera-related noise was configured to closely match CMOS cameras commonly used in LSCI applications (e.g. acA2040-90umNIR, Basler) with quantum efficiency $QE=0.45$ at 785 nm, read noise standard deviation 13$e^-$, pixel bit-depth of 8 bit, and saturation capacity 12,400$e^-$, but operating at 30% of it. Important to note that despite the sensor-average flux being 30% of the saturation capacity, some pixels might still appear saturated due to the skewness of the speckle intensity distribution. The dark current was set to 0 in all camera-related noise simulations, as it is generally irrelevant for LSCI due to relatively short exposure times. The MATLAB code used to generate the speckle images is available on GitHub^36^.

### In vivo imaging

For in vivo experiments, we have used N = 5 C57Bl6 mice (Janvier, Denmark weight, 12 weeks old) with chronically implanted cranial windows. All experimental protocols were approved by the Danish National Animal Experiments Inspectorate and were conducted according to the ARRIVE guidelines and guidelines from Directive 2010/63/EU of the European Parliament on the protection of animals used for scientific purposes. The animal preparation and the surgical procedure are standard and were described in detail previously^3,37^. Briefly, a craniotomy was performed, and an optically-transparent 4 mm round glass was installed in the mouse’s skull in the area of the barrel cortex. After the surgery, mice were allowed to recover for 5 days before imaging. Before starting surgical procedures or imaging sessions, the animals were anaesthetized in a chamber with 3% isoflurane mixed with oxygen at a flow of 1 L/min. During the surgery and imaging sessions, the mouse was placed on a servo-controlled heating table, maintaining their body temperature at 37 °C and the isoflurane concentration was reduced to a final concentration of 1–2%. Finally, when imaging, a holographic volume grating stabilized laser diode coupled to a polarisation-maintaining fibre (785 nm, Thorlabs FPV785P), controlled with a laser driver (Thorlabs LDC210C) and temperature controller (Thorlabs TED200C) was used to deliver coherent laser light on the cranial window. The scattered light was collected by VZM 450i zoom imaging lens (Edmund Optics) and recorded with a CMOS camera (Photron Nova S6, 20 × 20 $\upmu \text{s}^2$ pixel size, 6000 frames per second at 1024 × 1024 pixels) featuring large pixels (20 × 20 $\upmu \text{s}^2$) and high frame-rate necessary for this study.

Specifically, five recordings were made for each mouse. Three recordings were to test speckle-to-pixel size ratio effects, where the speckle-to-pixel size ratio was set to 2.4, 1.9 and 1.5 by adjusting the built-in aperture. A linear polariser was placed in front of the objective and adjusted in a “cross”-polarisation configuration to minimize specular reflections. To validate the effect of polarisation, two additional recordings were done in each mouse at the speckle-to-pixel size ratio of 1.5. For these recordings, the polariser was removed or positioned in a “parallel”-polarisation orientation. The average intensity on the camera was maintained at around 30% of saturation by introducing ND filters with different optical densities in the detection path. The five recordings were performed at the exposure time T = 1 ms (the longest exposure time available for the camera) and the corresponding frame rate of 1000 frames per second. To match the commonly used exposure time of 5 ms, before further analysis, the frames were decimated by a factor of 5 in time, using a moving average filter followed by downsampling. Finally, to test the effects of the exposure time on the LSCI precision measurement, an additional high-speed recording was performed in 3 of the animals. The frame rate was 20,000 frames per second, the exposure time T = 0.05 ms, the speckle-to-pixel size ratio was set to 1.5, and the polariser was in the “cross”-polarisation configuration.

### Data analysis

In computational and in-vivo experiments, the contrast was calculated as $K=\frac{\sigma (I)}{<I>}$, where $\sigma (I)$ and $<I>$ are the standard deviation and mean of the intensity calculated spatially over pixels neighbourhood. The neighbourhood size used in the analysis was 5 × 5 pixels unless stated otherwise. To quantify LSCI precision when analysing simulation results, we have calculated the mean relative per-pixel error as follows:

2 $$\begin{aligned} Error=\frac{1}{X*Y*N}\sum _{x,y,n}^{X,Y,N}\left |\frac{K(x,y,n)-<K>}{<K>}\right |*100\%, \end{aligned}$$

where *x*, *y* are indexes of pixel neighbourhoods over which *K*(*x*, *y*, *t*) was calculated, and *n* is the index representing statistically independent contrast images. $$<K>$$ is the contrast averaged across the entire data set. We excluded overlapping neighbourhoods—e.g. for the 5 × 5 neighbourhood, we have only used each 5th for the error calculation. To characterise LSCI signal quality in-vivo, we calculated the per-pixel signal-to-noise ratio (SNR) of the cardiac pulsation. The SNR was calculated by converting *K* to the blood flow index as $$BFI=\frac{1}{K^2}$$, calculating the Fourier spectrum for each pixel and measuring the respective amplitude of the cardiac activity peak compared to the noise plateau. We have used the median value of the SNR, which would represent signal quality in the parenchyma, for comparison between animals and conditions unless stated otherwise. Furthermore, to characterize the sharpness of the images when studying the effect of different polarisations, we have calculated image sharpness as an average of 3 × 3 spatial contrast calculated over the contrast image (i.e. measuring the average contrast of contrast). Only the pixels that maintained reasonable values in all time points of all recordings ($$0<I<255$$ and $$0.0001<K<0.99$$) were used to characterise the signal and image quality. Other pixels and their neighbourhood within 2 pixels were marked as artefacts and excluded from the analysis described above. When comparing the effect of different polarisations, the number of artefact pixels is reported in addition to other metrics. The error bars in all figures represent the standard deviation, and paired t-test was used for statistical analysis where relevant. The exact *p*-values, calculated with the t-test, except for *p*-values is below 0.001, which is reported as $$**p<0.001$$.

When analysing the exposure time data recorded at 20,000 frames per second, we identified the decorrelation time $\tau _c$ for each pixel using the Dynamic Light Scattering Imaging approach. A detailed procedure description can be found in our previous study^20^. Furthermore, we have used the contrast models derived in the follow-up study to explore the effect of the dynamics regime on absolute and relative contrast sensitivity. Specifically, we have used the following equations:

- Medium-sized vessels (multiple scattering ordered motion or single scattering unordered motion regimes)^16,18–20^:
  3 $$\begin{aligned} K =\beta ^{0.5}\bigg \{ \rho ^2 \frac{e^{-2x}-1+2x}{2x^2}+4\rho (1-\rho )\frac{e^{-x}-1+x}{x^2}+(1-\rho )^2\bigg \}^{0.5}+C. \end{aligned}$$
- Parenchyma or other capillary-perfused tissue (multiple scattering unordered motion)^16,20^:
  4 $$\begin{aligned} K =\beta ^{0.5} \bigg \{ \rho ^2 \frac{e^{-2\sqrt{x}}(4x+6\sqrt{x}+3)+2x-3}{2x^2}+8\rho (1-\rho )\frac{e^{-\sqrt{x}} (2x+6\sqrt{x}+6)+x-6}{x^2}+(1-\rho )^2\bigg \}^{0.5}+C. \end{aligned}$$
- Large vessels (single scattering ordered motion)^16,20^:
  5 $$\begin{aligned} K = \beta ^{0.5} \bigg \{\rho ^2\frac{e^{-2x^2}+\sqrt{2\pi }erf(\sqrt{2}x)x-1}{2x^2}+2\rho (1-\rho )\frac{e^{-x^2} +\sqrt{\pi }erf(x)x-1}{x^2} +(1-\rho )^2\bigg \}^{0.5}+C. \end{aligned}$$

In the Eqs. (3)–(5), *x* is the ratio between the exposure time *T* and decorrelation time $\tau _c$, $\rho$ denotes the portion of the dynamic scattering as opposed to the static scattering contribution ($1-\rho$), $\beta$ is the coherence degree parameter, and *C* is an offset caused by noise or insufficient speckle statistics^16–18,20^.

## Data Availability

The datasets generated during and/or analyzed during the current study are available from the corresponding author on reasonable request.
